# Educational outcomes of children with major congenital anomalies: Study protocol for a population-based cohort study using linked hospital and education data from England

**DOI:** 10.3310/nihropenres.13750.1

**Published:** 2024-11-06

**Authors:** Joachim Tan, Ayana Cant, Kate Lewis, Vincent Nguyen, Ania Zylbersztejn, Laura Gimeno, Pia Hardelid, Bianca De Stavola, Katie Harron, Ruth Gilbert

**Affiliations:** 1 NIHR Great Ormond Street Hospital Biomedical Research Centre, London, WC1N 1EH, UK; 2 University College London Great Ormond Street Institute of Child Health, London, WC1N 1EH, UK; 3 Centre for Longitudinal Studies, Social Research Institute, University College London, London, WC1H 0NU, UK

**Keywords:** Congenital abnormalities, birth defects, educational achievement, cohort study, school-aged children

## Abstract

**Background:**

Major congenital anomalies (CAs) affect around 2% of live births and are a primary cause of infant mortality, childhood morbidity and long-term disability, often requiring hospitalisation and/or surgery. Children with CAs are at greater risk of lower educational attainment compared with their peers, which could be due to learning disabilities, higher rates of ill-health and school absences, or lack of adequate educational support. Our study will compare the educational attainment of children with CAs to those of their peers up to age 11 in England, using linked administrative health and education data.

**Methods:**

We will analyse data from the ECHILD (Education and Child Health Insights from Linked Data) database. Children born in NHS-funded hospitals from 1st September 2003 to 31st August 2008 whose hospital records were linked to their educational records at three Key Stages (ages 4/5, 6/7 and 10/11 years) will be included. Children with different CAs, indicated by recorded hospital diagnosis codes, will be compared to children without CAs. We will compare the proportions of enrolled children who take the assessment, the proportions who reached national expected levels of attainment, and the mean standardised attainment scores for Maths and English at each Key Stage. We will describe variations in outcome by sex, ethnic minority background, region, and neighbourhood deprivation, and perform regression modelling to compare the attainment trajectories of children with and without CAs, controlling for sociodemographic factors.

**Ethics and dissemination:**

Ethics approval has been obtained for the analyses of the ECHILD database. Our findings will provide information for parents regarding their children’s expected academic potential, and also enable the development of interventions to support those at risk of not doing well. We will disseminate our findings to academics, policy makers, service users and providers through seminars, peer-reviewed publications, conference abstracts and other media (lay summaries and infographics).

## Plain language Summary

Major congenital anomalies (CAs hereafter), also known as birth defects, occur during pregnancy and affect around 2% of babies born in England. Children with CAs experience significant ill-health and undergo hospitalisation and surgery, which contribute to increased school absences. Some CAs, such as Down Syndrome, are also associated with learning disabilities. Compared with their peers, children with CAs are at greater risk of not reaching expected levels of academic attainment for their age which, in turn, can have a detrimental effect on their development and quality of life.

Using health and education data of children born in England between 2003–2008, we will compare the academic performance of children with and without CAs at different stages of education in state-funded schools (ages 4/5, 6/7 and 10/11 years). We will also examine how results vary by sociodemographic factors. The study data are pseudonymised, meaning that they do not contain personally identifiable information, and are stored in a secure environment with access restricted to the project's researchers only.

Improving our understanding of the educational achievement of children with CAs will enable parents, carers, and teachers to form positive and realistic expectations regarding the children's needs and prospects, to identify barriers to learning, and to inform strategies for helping those at risk of not doing well. Through this, children with CAs can be supported to reach their full potential and enhance their future life chances.

## Introduction

Congenital anomalies, also known as birth defects, are structural, chromosomal or genetic disorders which occur during fetal development. Major CAs (hereafter CAs for simplicity) are those with significant medical, functional or social consequences for individuals, and collectively they affect about 2% of live births in England (10,119 in 2021) ^1^. CAs are a major cause of increased mortality during infancy, morbidity in childhood and long-term disability ^2^. Medical and surgical developments have contributed to improvements in the survival of children with CAs over time, leading to more of these children reaching school age ^3^. Children with CAs have more complex health needs, often requiring hospitalisation for surgery ^4, 5^. Ill-health and higher rates of school absences, combined with inherent learning disabilities in some types of CAs, are likely to adversely impact the learning and school achievement of affected children ^6, 7^. There is evidence that children with CAs such as cardiac defects, orofacial clefts or spina bifida, are at higher risk of lower academic attainment and are more likely to receive Special Education Needs (SEN) provision than children without CAs ^8^. Such findings were corroborated by a recent study using data from regional CA registries linked to national educational data, which found that proportionally fewer children with a range of non-syndromic CAs achieved expected levels of educational attainment at ages 11 and 16 in England compared with their peers ^9^.

This study is part of the Health Outcomes of young People in Education (HOPE) research programme, which aims to explore variation in SEN provision and understand its impact on health and education outcomes using linked administrative data ^10^. We will analyse data on all children with and without CAs in the whole population and track their attainment trajectories through primary school from ages 4 to 11. Understanding which children may not reach expected levels of attainment will, in turn, help to inform the prioritisation of interventions that are integrated across sectors, and aim to improve the educational outcomes of those most in need of support. Furthermore it would address the information needs of parents of children with CAs, who seek more accurate and affirmative messages about their children’s quality of life and intellectual development, and help them plan for appropriate forms of support as needed ^11, 12^.

### Aim and objectives

This study aims to describe the educational attainment of children born with CAs over time, and how these vary according to their specific CA and other characteristics. The specific objectives are to:

1. Develop cohorts of children with specific CAs including nervous system anomalies, orofacial clefts, cardiac defects, gastrointestinal, renal or limb anomalies, and chromosomal syndromes, using diagnosis codes from hospital records and reference code lists.
2. Describe the educational outcomes of children with and without CAs, in terms of whether they sat exams, reached nationally expected levels of attainment and their standardised subject test scores, at three Key Stages of primary education (Early Years Foundation Stage (EYFS), Key Stage 1, Key Stage 2, corresponding to ages 5, 7, 11 respectively).
3. Compare trajectories in educational attainment over time for children with and without CAs, unadjusted and adjusting for sociodemographic factors (sex, ethnicity, deprivation).

## Methods

### Patient and Public Involvement

Previous research with parents and carers of children with CAs across Europe (including the UK) have shown that they wanted more information about their children’s intellectual development and full potential, and support with school and education ^12^. The HOPE study team conducted meetings in 2020 and 2021 with patient, pupil, and public engagement groups. These groups included the Great Ormond Street Hospital Young Persons’ Advisory Group for research (YPAG), Council for Disabled Children’s Group (FLARE), and the National Children’s Bureau Families Research Advisory Group (FRAG). The FLARE group desired more awareness of linked administrative data (such as the ECHILD database) in research to better support similarly affected children. Attitudes were positive towards the design and conduct of the HOPE study, with emphasis being placed on the importance of covering the whole population to investigate the interrelated areas of health and education. Feedback obtained from YPAG in November 2021 also showed consensus on exams and performance in school being stressful for children with chronic health conditions, which led us to frame educational attainment as a study objective. Additional meetings with these groups also underlined the importance of continued recruitment of the public to embed children and young people’s voices in the way our findings are interpreted and translated. Key learnings from past public engagements can be found here.

### Ethics and dissemination

Permissions to use linked, de-identified data from Hospital Episode Statistics and the National Pupil Database were granted by the Department of Education (DR200604.02B) and NHS Digital (DARS-NIC-381972). Ethical approval for the ECHILD project was granted by the National Research Ethics Service (17/LO/1494, dated 26 Sep 2017), NHS Health Research Authority Research Ethics Committee (20/EE/0180, dated 10 Jul 2020), and UCL Great Ormond Street Institute of Child Health’s Joint Research and Development Office (20PE16, dated 02/10/2020). Participant consent is not required as the data are anonymised and contains no personally identifiable information. Access to the ECHILD database is approved by the ECHILD team ( ich.echild@ucl.ac.uk) and data can only be used within the Office for National Statistics Secure Research Service by approved researchers.

Findings will be disseminated via peer-reviewed publications, conference presentations, the ECHILD website, seminars, and workshops. The target audience will comprise various stakeholders including academics, patient and public representatives, health and social care professionals, teachers, and policy makers. Methods and final code (including scripts to define populations, exposure, outcomes and covariates) will be made available through publications and the ECHILD code repository ( https://code.echild.ac.uk/), to facilitate the reproducibility and extension of our analyses.

### Study design, setting and population

This is a population-based cohort study that will use linked administrative data from hospital admissions and educational databases in England.

### Data source

The ECHILD (Education and Child Health Insights from Linked Data) database contains routinely-collected healthcare data from Hospital Episode Statistics (HES) linked to educational data from the National Pupil Database (NPD) for children born in England from 1995 onwards, equating to approximately 14.7 million individuals up to 2020 ^10, 13^. HES contains information on NHS-funded hospital admissions in England, including length of stay, diagnoses and procedures performed, demographic and geographical information; data on outpatient consultations, accident and emergency visits and critical care were successively added in later years, but do not cover the entire period of our study. HES has been routinely linked to ONS mortality records, including information on causes and timing of deaths, since 1998. HES captures about 97% of births in NHS-funded hospitals in England and 98–99% of all secondary care contacts ^14^. In HES, diagnostic codes and causes of death are coded using the International Classification of Diseases 10 ^th^ Revision (ICD-10). Procedure codes are coded using the Office of Population Censuses and Surveys Classification of Interventions and Procedures 4th Revision (OPCS-4).

The NPD consists of several datasets which capture information on school registration, attainment, absences and exclusions for all children attending state-funded schools in England. Data on educational attainment include national tests scores and teacher-assessed attained levels at different time points (referred to as Key Stages) between the ages of 4 to 18 years. Information on pupil enrolment is collected in the School Census three times every academic year since 2007/8 (Autumn, Spring and Summer terms) and include pupil’s ethnicity, Special Education Needs (SEN) provision, area-level deprivation indices, free school meals eligibility (FSME) and school identifier, which can be used to indicate the type of school (e.g. mainstream or special schools).

The records in HES and NPD were deterministically linked by NHS England through an algorithm which uses real-world identifiers (including name, date of birth, sex, and postcode) but these identifiers do not exist in ECHILD; instead pseudonymised ‘meaningless’ keys were created to link information belonging to each unique individual for research purposes. This enables the ECHILD database to be used for longitudinal studies aiming to evaluate the health and educational trajectories of various groups of children. ECHILD does not include individuals who have notified NHS that their information should not be shared for purposes unrelated to their direct care (e.g. research). Further details can be found in previously published data profiles and related study protocols ^10, 13, 15^.

### Study population

**Figure 1.**
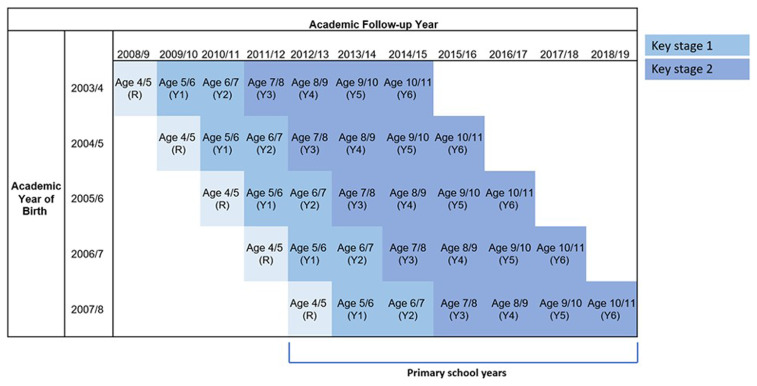
Expected age at each year of primary school by birth year and follow-up year. R = reception. Y = year. Birth and follow-up year defined according to the academic calendar (e.g. 2003/4 includes 1 ^st^ September 2003 to 31 ^st^ August 2004, inclusive). *This figure has been reproduced with permission from Cant A, Zylbersztejn A, Gimeno L et al. Primary school attainment outcomes in children with neurodisability: Protocol for a population-based cohort study using linked education and hospital data from England [version 1; peer review: 1 approved with reservations]. NIHR Open Res 2024, 4:28 ( https://doi.org/10.3310/nihropenres.13588.1) ^15^*.

The study population includes all singleton children born in NHS-funded hospitals between 1st September 2003 and 31st August 2008 who were linked to NPD and enrolled in Reception (typically at age 4/5) in academic years 2007/08 to 2012/13. We include all pupils enrolled in state-funded mainstream schools, special schools, maintained pupil referral units or alternative provision based on records in the Spring census (taken in January following the start of the academic year the previous September), Alternative Provision Census or Pupil Referral Unit Census (both collected in January). The Spring School Census is used to capture our study population as it is the basis for the allocation of school funding and is assumed to be the most complete of the three censuses in each academic year. The birth years were chosen to ensure that the youngest children (born academic year 2007/8) could be expected to have completed primary school (end of Year 6, aged 10/11) by 31st August 2019 ( Figure 1). This was the last complete academic year of follow-up before the COVID-19 pandemic, which led to disruption and discontinuity in school attendance, assessments and educational records ^16, 17^. Children who are two or more years outside of the expected age for their school year, which is expected to be a very small number, will be excluded.

The follow-up period will be from the start of Reception until the end of Year 6. We will track children’s educational attainment by measuring assessment outcomes at the end of Reception (aged 4/5), Year 2 (aged 6/7) and Year 6 (aged 10/11). Children who do not appear in all three of the Spring Censuses (i.e. not enrolled) will be excluded from the analyses. Reasons for loss to follow-up include transition to non-state funded education, emigration, death, or other causes. We will enumerate the number of pupils excluded at each Key Stage and compare them with included children to evaluate the potential impact on study results.

### Major Congenital Anomalies (CAs)

Our main comparison will be between children with any CA to children without CAs (peers). We will additionally compare subgroups of children with non-chromosomal, non-genetic CAs (isolated CAs; see below), and children with selected chromosomal or genetic CAs, to their peers. CAs will be indicated by specific ICD-10 diagnosis codes and/or OPCS-4 procedure codes recorded in HES, as well as ICD-10 codes in death registrations. We will include diagnoses recorded in the first year of life or causes of death at any age.

### Phenotyping CAs

The EUROCAT (European network of population-based registries for the epidemiological surveillance of congenital anomalies) classification system contains code lists for assigning CA subgroups based on clinically-validated ICD-9 or ICD-10 diagnostic codes (with the British Paediatric Association (BPA) extension) recorded for each CA case by member registries ^18^. The EUROCAT codes were found to identify more children with CAs in administrative data compared with other code lists for identifying children with chronic conditions (including CAs), such as Hardelid and Feudtner codes, as discussed in Zylbersztejn *et al.* ^19– 21^. We will phenotype CAs using the code list in EUROCAT guide version 1.4 ^22^, organising CAs into subgroups based on organ system affected (nervous system, cardiac, respiratory, gastro-intestinal, abdominal wall, renal, limb etc.). Subgroups defined by 5-digit ICD-10 BPA codes which are not present in HES, teratogenic syndromes, and CAs resulting from maternal infections, will be excluded from our study.

The application of EUROCAT code lists – developed for member registries who perform specialist clinical coding and validation of CAs – directly to administrative data such as HES for defining exposures, may result in missed or false positive cases. This could be due to temporal hospital coding variations but can also result from differential diagnoses or CAs with variable severity (e.g. atrial septal defects, congenital hydronephrosis). A European study (including data from England and Wales) examining the coding of CAs in hospital databases found that the sensitivity and specificity of diagnoses (termed completeness and validity respectively) varied by type of CA ^23^. Hence where available, we will augment our approach using methods (mostly combining diagnostic and procedure codes) that have been developed for HES data for a subset of CAs ^24^. A summary of these CAs and methods is presented in Table 1. We will compare the prevalence of CA subgroups ascertained by our methods to published statistics, including EUROCAT prevalence tables and estimates from related published studies.

**Table 1.** Congenital anomaly (CA) subgroups and published identification methods developed for Hospital Episode Statistics (HES) data.

CA subgroups [ICD-10 code(s)]	Summary of method	Source article
Severe congenital heart defects [Q200, Q201, Q203, Q204, Q212, Q213, Q220, Q224, Q225, Q226, Q230, Q232, Q233, Q234, Q251, Q252, Q262]	Diagnosis code AND/OR Procedure code indicative of cardiac surgery and therapeutic interventional catheterisation procedures AND procedure was : a. Performed in paediatric cardiac centre b. Not standalone intervention for patent ductus arteriosus in birth <37 weeks gestation or birthweight <2500g	Gimeno *et al.*, 2023 ^26^
Cleft palate [Q35] ^a^; cleft lip [Q36]; cleft palate with cleft lip [Q37] ^b^	Diagnosis code AND procedure code for primary cleft repair (OPCS-4: F031 or F291).	Fitzsimons *et al.*, 2017 ^27– 30^
Anorectal malformations [Q420, Q421, Q422, Q423, Q435, Q436, Q437, Q522, K604, K605, K624, N321, N360, N823, N824, Q438, Q439] ^d^	Diagnosis code in hospital OR death record AND repair code; OR Infants with diagnosis code in death record AND supportive diagnosis code in hospital record	Ford *et al.*, 2021 ^31^
Congenital diaphragmatic hernia [Q790]	Diagnosis AND repair codes, OR recorded as cause of death. Infants with diagnosis OR repair codes only would require relevant supportive codes. Cases with codes indicating potential misdiagnosis or associated malformations were excluded.	Peppa *et al.*, 2023 ^32^
Hypospadias [Q54] ^c^	Procedure code for primary repair of hypospadias (OPCS-4: M731) with or without a diagnostic code, excluding concomitant diagnosis codes [ICD-10: Q560-Q564, E250, E258, E259, E345, Q640, Q641].	Wilkinson *et al.*, 2017 ^33^

CA: congenital anomaly; HES: Hospital Episode Statistics; ICD-10: International Classification of Diseases 10th Revision; OPCS-4: Office of Population Censuses and Surveys Classification of Interventions and Procedures 4th Revision. ^a^EUROCAT excludes Q357 as a minor anomaly. ^b^Associations with holoprosencephaly (Q042) or anencephaly (Q00) are excluded from all EUROCAT orofacial clefts subgroups. ^c^EUROCAT excludes Q544 as a minor anomaly. ^d^Additional differential diagnosis codes were used to exclude cases unlikely to have anorectal malformations.

### Isolated and multiple CAs

Children with a single structural CA, or multiple CAs occurring in one organ system only (e.g. VSD with pulmonary stenosis) are considered to have an isolated CA. Where CAs occur in more than one organ system (e.g. orofacial clefts with heart defects) which are not part of a known sequence (additional anomalies caused by a primary structural CA, such as Pierre Robin sequence), they may be linked to chromosomal or genetic malformations, or have an external or unknown aetiology. We will follow the EUROCAT flowchart for the classification of children into chromosomal, genetic, or isolated CA groupings (neural tube, cardiac, renal, other), with the residual unclassified group being assigned to “potential” multiple CAs ^25^. For our study, the primary analysis will be on children with isolated, chromosomal or genetic CAs, as their results will be representative with regard to the specific CA. Due to their relative heterogeneity, results for those with potential multiple CAs will mainly serve to check that our findings are consistent with expectations of poorer achievement on average than children with isolated CAs.

## Outcomes: Educational attainment

At the end of Reception year (age 4/5), Year 2 (age 6/7) and Year 6 (age 10/11), corresponding to Early Years Foundation Stage (EYFS), Key Stage 1 and Key Stage 2 respectively (collectively referred to as Key Stage (KS) henceforth), children are assessed whether they have reached National Curriculum (NC) expected levels in particular areas of learning. These may be based on teachers’ assessments, scores from tests taken or a combination of both. We will compare the following outcome measures between exposure groups defined above:

- Proportion of children who were enrolled and did not complete the assessment for each KS.
- Of those who were assessed, the proportion of children who reached NC expected attainment levels.
- Cohort-specific standardised scores for Reading and Maths (or equivalent areas for EYFS) derived from raw scores of all pupils taking the tests in a given academic year.

Reception: Early Years Foundation Stage Profile (EYFSP)

The EYFSP is a report consisting of teachers’ assessments of children’s development across several learning domains at the end of Reception, to support their transition to Year 1. Until academic year 2011/12, children were scored (0–9) in 13 assessment scales grouped into 6 areas of learning, where a score of ≥6 was deemed the expected standard. From 2012/13 onwards the EYFSP was changed to a grading of “emerging”, “expected” or “exceeding” for each 17 Early Learning Goals (ELGs) across 7 areas of learning. A ‘Good Level of Development’ (GLD) is attained when the child has attained sufficient scale points in requisite areas (pre-2012/13) or reached expected or higher levels in the ELGs in the following prime areas of learning: personal, social, and emotional development; communication and language; physical development; mathematics; and literacy (2012/13 onwards) ^34^. We will use the GLD as the indicator for reaching the expected level of attainment at EYFS.

Year Two and Year Six: Key Stage Assessments

At the end of KS1, pupils were awarded NC levels for Reading and Maths based on teacher assessments, with level 2B being the expected standard of attainment. These levels were also converted to point scores to enable inter-KS comparisons ^35^. For KS2, pupils sit for externally marked tests, with maximum raw scores in Reading and Maths being 50 and 110 (100 before 2015/16) respectively ^15^. NC levels were also awarded to indicate whether children were below, at, or above the expected level (Level 4) for the subject. For our study, we will analyse whether pupils achieved expected levels of attainment (binary) and standardised scores (calculated using the mean and standard deviation of the raw scores of all pupils who sat the tests in a given academic year) as outcomes. These are to reflect changes in the recording of educational outcomes across years, other period effects (e.g. Flynn effect) ^36^, as well as to enable comparisons with previous studies using similar measures ^37, 38^. Table 2 summarises information on educational outcomes.

**Table 2.** Summary of Educational Outcomes.

National Curriculum (NC) Year Group	Typical pupil age, years	Key Stage	Academic Years analysed	Measures of Attainment
Reception	4/5	EYFS	2008/09 to 2012/13	• Communication, Language and Literacy; Problem Solving, Reasoning and Numeracy; Total EYFSP scores • Good Level of Development
Year 2	6/7	KS1	2010/11 to 2014/15	• Point Scores in Reading and Maths • Achieved expected NC level in Reading and Maths
Year 6	10/11	KS2	2014/15 to 2018/19	• Scores for Reading and Maths • Achieved expected NC level in Reading and Maths

EYFS: Early Years Foundation Stage. EYFSP: Early Years Foundation Stage Profile. KS: Key Stage. NC: National Curriculum.

### Additional variables

We will use data from HES and NPD to derive additional variables to characterise children in the cohort and include in statistical models:

1) Sex at birth as recorded in HES (henceforth sex).
2) Relative month of birth: the youngest children in each school year (born in August) have lower average attainment compared to older peers (the August-September gap) ^39^.
3) Maternal age at birth, in years: this will be obtained from the birth record of the child in HES.
4) Modal recording of child’s major ethnic group in NPD school censuses (Asian, Black, Chinese, Mixed, White, Other, or unclassified).
5) Income deprivation affecting children index (IDACI) quintile. This measures the proportion of children under the age of 16 within a Lower layer Super Output Area who are living in an income-deprived household ^40^.
6) Free school meals eligibility (FSME) (yes/no).
7) Government Office Region of residence associated with the pupil’s residential address (North East, North West, Yorkshire and the Humber, East Midlands, West Midlands, East of England, London, South East, South West, or missing).

For variables 5, 6, 7 we will use the earliest non-missing value recorded in the NPD school censuses.

### Descriptive analysis

Derivation of the analysis sample will be depicted using a flowchart, showing the number of included and excluded children with each application of the selection criteria. We will describe follow-up and loss to follow-up in children with and without CAs (i.e. the proportion of children not enrolled at Reception, Year 2, Year 6) and the percentage of children who died before the end of primary school (age 10/11), using HES-ONS linked mortality data in ECHILD. For each CA subgroup and children without CA, we will describe (1) the proportion of enrolled children who were not assessed at each timepoint; (2) the proportion of assessed children who reached nationally expected levels; and (3) mean standardised scores for EYFSP, KS1 and KS2 assessments. Results will be stratified by sex and academic year.

### Statistical analysis

We will examine trajectories of children’s attainment using longitudinal data from the three stages of education (EYFS, KS1 and KS2). Linear mixed models will be fitted to repeated subject-specific standardised scores, with explanatory variables that include an indicator of school year, whether the child was born with a specific CA, and their interaction. Alternative models that include only random intercepts or both random intercepts and slopes will be compared to account for individual variability in average score over time and in the rates of change over time. Generalised linear mixed models will be used to analyse trajectories in reaching the expected levels of attainment (binary outcome) at each KS, comparing those with CAs to those without, using the same modelling steps. We will report results from unadjusted analyses and adjusted models controlling for birth and sociodemographic variables outlined previously, to examine the extent to which other factors could explain any observed associations.

### Missing data

For region and relative month of birth, if data are missing in Reception, we will use the earliest complete recording available in any subsequent January school census under the premise that these characteristics do not change over time. For effect estimation, we will explore different approaches depending on the variables and extent of missing data, such as comparing results using best-worst or min-max scenarios, or multiple imputation using chained equations (under the assumption that missingness is Missing At Random) ^41^.

### Potential bias

Our aim is to compare children with major CAs to those without CAs, rather than to “healthy” children. Consequently, some children in the unaffected reference group will have other health conditions that may have an unmeasured influence on attainment outcomes. Second, as described above, misclassification may occur in indicating children with CAs. The relative lack of specificity in ICD-10 in HES, coupled with the possibility of approximate or incorrect diagnosis codes, may generate false positives or negatives for a subset of CAs. Third, children with CAs that do not require inpatient care or surgery may be misclassified into the unaffected group, which can bias group differences towards the null, leading to estimates that are likely to be conservative. To comprehensively address these issues would take us beyond the scope of the current study, but we propose to examine the impact of varying the inclusion criteria for diagnosis codes in the following ways:

1) Including diagnosis codes at any age until the end of primary school (31st August of the year the child attends Year 6).
2) Excluding incomplete diagnosis codes (2 or 3 characters) and selected “unspecified” codes, on the hypothesis that these may be tentative, unconfirmed observations or minor anomalies ( Table 3).
3) Excluding EUROCAT codes which are not also present in the Hardelid or Feudtner code lists.
4) Including diagnosis codes from HES outpatient contacts (national coverage expected for births =2005).

**Table 3.** List of non-specific ICD-10 codes proposed for exclusion in sensitivity analyses.

ICD-10 code	Description
Q049	Congenital malformation of brain, unspecified
Q079	Congenital malformation of nervous system, unspecified
Q159	Congenital malformation of eye, unspecified
Q179 ^1^	Congenital malformation of ear, unspecified
Q189 ^1^	Congenital malformation of face and neck, unspecified
Q249	Congenital malformation of heart, unspecified
Q289	Congenital malformation of circulatory system, unspecified
Q309	Congenital malformation of nose, unspecified
Q319	Congenital malformation of larynx, unspecified
Q349	Congenital malformation of respiratory system, unspecified
Q386	Other congenital malformations of mouth
Q459	Congenital malformation of digestive system, unspecified
Q529	Congenital malformation of female genitalia, unspecified
Q559	Congenital malformation of male genital organ, unspecified
Q649	Congenital malformation of urinary system, unspecified
Q749	Unspecified congenital malformation of limb(s)
Q799	Congenital malformation of musculoskeletal system, unspecified
Q898	Other specified congenital malformations
Q899 ^1^	Congenital malformation, unspecified
Q999	Chromosomal abnormality, unspecified

^1^already excluded as a minor anomaly code in EUROCAT; listed here for completeness

## Data Availability

No data are associated with this article.
