# Supplementary material for: Educational outcomes of children with major congenital anomalies: Study protocol for a population-based cohort study using linked hospital and education data from England
Source: NIHR Open Res. 2024 Nov 6;4:68. [Version 1] doi: 10.3310/nihropenres.13750.1 (PMC11803390; doi:10.3310/nihropenres.13750.1)
Supplement: Peer review history

# Reviewer report: Reviewer response for version 1

**DOI:** 10.3310/nihropenres.14936.r34175

**Published:** 2025-02-07

**Authors:** Jennifer Broughan

**Affiliations:** 1 National Disease Registration Service, Leeds, UK

This article describes a protocol for a study that aims to assess differentials in educational attainment between children with and without CA, in England and for children born in NHS hospitals from September 2003 to August 2008.

 The protocol and the study it proposes are of high importance to understand inequalities in educational attainment for children. The protocol is clear and detailed and it offers a good description of the background, aims, objectives and limitations.

 A limitation that deserves more discussion  is  the applicability of using administrative data where better alternatives  exist eg congenital anomaly registers. HES is a valuable data source but the quality of the clinical coding for some of these extremely rare conditions remains unknown and this should be caveated appropriately.  More information on the granularity the sub groups planned for analysis is needed. EUROCAT subgroups (cardiac, respiratory etc) can be heterogenous mix of severity.  Limiting to diagnoses  recorded in  the first year of life will exclude many conditions where surgery is deferred or multi-staged. Within EUROCAT subgroups, some conditions are repaired at birth and some will require life time care. Using OPCS codes will improve the specificity of the codes but this will result in further inequities in data quality across anomaly groups. Excluding the unspecified ICD codes, will improve the specificity of HES coding but may result in losing large number of true cases. There is also a risk that children with structural anomalies will later be found to have developmental delay or genetic syndromes that won’t be recorded in HES and may impact their educational attainment in ways that an isolated structural anomaly would not be expected to have an impact. 

 There was no nationwide overage of congenital anomaly registration at the time but there was regional coverage and sensitivity analysis of the results using known CA cases  would be a possible and useful exercise.

 The expected output is ambitious, considering the intricacies of the comparison between children with CA and their peers without CA when moving down to the details of EUROCAT CA groups. Aim 1 sets out to define cohorts also by CA group, but Aims 2 and 3 only mention differences between children with and without CAs. It is not clear whether after the descriptive analysis (which will present distributions of educational attainment for each major EUROCAT CA group), the modelling part will keep this level of detail or not. Longitudinal growth model such as those proposed in this protocol require at least 3 time/measurement points for each child, and I wonder whether children with certain (more severe) CA will end up being underrepresented by these models. Also, the generalised LMM for trajectories are mentioned for comparison between children with CAs versus those without CAs, without reference to CA groups.

 The article is generally sound and of high importance, hence I would recommend indexing after clarifications on the suitability of the methods proposed for the comparisons across EUROCAT CAs groups, if that is the main aim.

Is the study design appropriate for the research question?

Partly

Is the rationale for, and objectives of, the study clearly described?

Yes

Are sufficient details of the methods provided to allow replication by others?

Partly

Are the datasets clearly presented in a useable and accessible format?

Yes

Reviewer Expertise:

Statistical modelling; public health; education; congenital anomalies; inequalities, epidemiology.

I confirm that I have read this submission and believe that I have an appropriate level of expertise to confirm that it is of an acceptable scientific standard, however I have significant reservations, as outlined above.

# Reviewer report: Reviewer response for version 1

**DOI:** 10.3310/nihropenres.14936.r34057

**Published:** 2025-01-02

**Authors:** Kirti Naranje

**Affiliations:** 1 Sanjay Gandhi Postgraduate Institute of Medical Sciences, Lucknow, India

Introduction:

 This study addresses an essential but underexplored area, linking congenital anomalies (CAs) with educational attainment. Its strengths lie in leveraging population-wide linked administrative data and focusing on specific CAs to provide nuanced insights. However, the reliance on hospital diagnosis codes may underrepresent milder or undiagnosed cases, potentially skewing results. The study could benefit from a qualitative dimension to capture lived experiences of children and families, complementing quantitative findings. Including qualitative insights or targeted interventions based on findings would strengthen the applicability of results for educators and policymakers.

 Methods :

- The exclusion of non-state school attendees and children with incomplete data could introduce selection bias, limiting generalizability.
- Reliance on hospital-coded data may lead to underreporting or misclassification of congenital anomalies, impacting subgroup analyses
- Using administrative data for longitudinal tracking is valuable, but loss to follow-up due to emigration or other factors could skew results.
- The study's focus on pre-COVID-19 data avoids pandemic-related disruptions but misses insights into how external shocks might exacerbate disparities.
- The reliance on teacher assessments for EYFSP and KS1 may introduce subjective biases, potentially affecting validity.
- Variables like FSM eligibility and IDACI quintiles provide a socio-economic lens, but regional and temporal variations in their impact may warrant further exploration.
- Statistical analysis:
- The authors have very well described statistical methods as well as how they will handle missing data and potential biases

Is the study design appropriate for the research question?

Yes

Is the rationale for, and objectives of, the study clearly described?

Yes

Are sufficient details of the methods provided to allow replication by others?

Yes

Are the datasets clearly presented in a useable and accessible format?

Not applicable

Reviewer Expertise:

Neonatology

I confirm that I have read this submission and believe that I have an appropriate level of expertise to confirm that it is of an acceptable scientific standard.

# Reviewer report: Reviewer response for version 1

**DOI:** 10.3310/nihropenres.14936.r33447

**Published:** 2024-12-26

**Authors:** Michael Fleming

**Affiliations:** 1 University of Glasgow, Glasgow, Scotland, UK

In short I think this is an excellent protocol paper, written in a very clear manner with very clear and robust methods. I have no issues with the submission. The rationale for the project are clearly laid out and the research questions are appropriate and clear. The methods of ascertainment to identify children with different types of anomalies are described clearly and several different code lists (involving ICD and OPCS codes) are discussed and considered including EUROCAT methods and lists by Hardelid and Feudter. Measures of educational attainment are also clearly explained and seem appropriate and feasible to examine within the ECHILD data. The overall study cohort (timelines and follow up years etc) also seems appropriate and fits with available data in ECHILD. The statistical analyses seem robust and appropriate and the authors have taken care not only to outline some limitations and potential biases resulting from the study design and available data, but have also outlined how they plan to account for those by for example varying inclusion criteria etc. Overall, this is an excellent paper and I feel the project has real potential to provide impactful findings.

Is the study design appropriate for the research question?

Yes

Is the rationale for, and objectives of, the study clearly described?

Yes

Are sufficient details of the methods provided to allow replication by others?

Yes

Are the datasets clearly presented in a useable and accessible format?

Yes

Reviewer Expertise:

I am a senior lecturer in public health and chartered statistician with 20 years experience working in public health analysing population-wide linked health data and over ten years experience of analysing population-wide linked education data.  My background is in statistics, epidemiology, data linkage and data science and my research focusses on maternal and child outcomes including health, educational and neurodevelopmental outcomes in children related to early life and obstetric/maternity factors.

I confirm that I have read this submission and believe that I have an appropriate level of expertise to confirm that it is of an acceptable scientific standard.

# Reviewer report: Reviewer response for version 1

**DOI:** 10.3310/nihropenres.14936.r33453

**Published:** 2024-11-28

**Authors:** Melanie Ehrler

**Affiliations:** 1 Forensic and Neurodevelopmental Sciences, King's College London Institute of Psychiatry Psychology & Neuroscience, London, England, UK; 2 University Children's Hospital Zurich, Zurich, Switzerland

I congratulate the authors on this very interesting and important project which will certainly be highly informative for research, health care professionals and most importantly for the patients and their families. I particularly appreciate the patient involvement to define the research questions, the longitudinal design and the transdiagnostic approach. However, I have a few comments that could be clarified in this protocol prior to data analysis.

 Overall, I suggest defining specific hypotheses about the direction of effects based on previous literature (not just definition of overall aims). I would be particularly interested if the authors can formulate a specific hypothesis about the slope of the trajectory of educational attainment in children with CA? Do you expect educational difficulties to increase, decrease or remain stable over time in children with CA - based on previous literature?

 The authors plan to compare children with syndromic vs. isolated CA. However, I suggest conducting additional comparison between different types of CA. This may be of importance as different CAs may have different effects on brain development and eventually influence neurodevelopmental outcomes and educational attainment. For instance, several studies have shown that children with congenital heart disease demonstrate a wide range of brain alterations, partially due to hypoxia and altered cerebral blood perfusion. Consequently, these children have high rates of neurodevelopmental and educational difficulties. It is unclear whether other CAs (e.g., gastro-intestinal) have a similar risk for neurodevelopmental and educational difficulties. Stratifying the analyses based on the type of CA may help interpret the findings of this study.

 Also, different types of CA may have varying clinical courses. There may be differences in school absence due to hospital visits depending on the type of CA. Considering that the aim of this study is to better inform patients and their families, some information about disease-specific outcomes should be provided. The large sample size of this study may allow for these additional (exploratory) analyses.

 This study includes children born between 2003 and 2008, however ECHILD includes data from 1995 until 2020. The authors justify the selection of the latest inclusion timepoint (born 2008, completing primary school prior to COVID pandemic). However, they do not justify why they only consider children born from 2003 and not earlier.

 It is unclear why children, who are two or more years outside of the expected age for their school year, are excluded. Excluding these children may limit the generalizability of the findings. Also, this subgroup may be particularly vulnerable and worth investigating. Please justify your decision.

 Why did the authors decide to conduct complete case analysis (complete educational data at all three measurement time points)? Did the authors consider imputation methods? Considering that children with complex CA may be absent from school more often, these children may have a higher likelihood of missing data.

 The degree and timing of SEN provision allocated to a child may likely influence their longitudinal educational outcome (e.g., SEN x time interaction). I suggest that the authors define in their protocol how this is statistically handled. It would be interesting to see whether SEN provision is associated with sociodemographic factors and/or type of CA (e.g. the “visibility” of the CA may influence if a child receives SEN provision more easily).

Is the study design appropriate for the research question?

Partly

Is the rationale for, and objectives of, the study clearly described?

Yes

Are sufficient details of the methods provided to allow replication by others?

Partly

Are the datasets clearly presented in a useable and accessible format?

Yes

Reviewer Expertise:

Neurodevelopmental outcomes of children born with medically complex conditions.

I confirm that I have read this submission and believe that I have an appropriate level of expertise to confirm that it is of an acceptable scientific standard, however I have significant reservations, as outlined above.
